# A comparison of qPCR and ddPCR used for quantification of the *JAK2* V617F allele burden in Ph negative MPNs

**DOI:** 10.1007/s00277-018-3451-1

**Published:** 2018-07-28

**Authors:** Dorota Link-Lenczowska, Niels Pallisgaard, Sabrina Cordua, Magdalena Zawada, Sylwia Czekalska, Dorota Krochmalczyk, Zuzanna Kanduła, Tomasz Sacha

**Affiliations:** 1 Department of Hematology Diagnostics, The University Hospital, Kraków, Poland; 2 Department of Pathology, Zealand University Hospital, Roskilde, Denmark; 3 Department of Hematology, Zealand University Hospital, Roskilde, Denmark; 4 Chair of Hematology, Jagiellonian University Medical College, Kopernika 17, 31-501 Krakow, Poland; 5 Department of Hematology and Bone Marrow Transplantation, Poznan University of Medical Sciences, Poznan, Poland

**Keywords:** Myeloproliferative neoplasms, JAK-inhibitor, qPCR, ddPCR, Limit of detection, Minimal residual disease

## Abstract

Philadelphia-negative myeloproliferative neoplasms (MPNs) are a diverse group of diseases whose common feature is the presence of V617F mutation of the *JAK2* gene. In the era of novel therapeutic strategies in MPNs, such as JAK-inhibitor therapy, there is a growing need for establishing high sensitive quantitative methods, which can be useful not only at diagnosis but also for monitoring therapeutic outcomes, such as minimal residual disease (MRD). In this study, we compared the qPCR and ddPCR methods and their clinical utility for diagnosis, prognostication, and treatment monitoring of MPNs with *JAK2* V617F mutation in 63 MPN patients of which 6 were subjected to ruxolitinib treatment. We show a high conformance between the two methods (correlation coefficient *r* = 0.998 (*p* < 0.0001)). Our experiments revealed high analytical sensitivity for both tests, suggesting that they are capable of detecting the *JAK2* V617F mutation at diagnosis of MPN with a limit of detection (LoD) of 0.12% for qPCR and 0.01% for ddPCR. The alterations of *JAK2* V617F allele burden in patients treated with ruxolitinib were measured by both methods with equal accuracy. The results suggest an advantage of ddPCR in monitoring MRD because of allele burdens below the LoD of qPCR. Overall, the clinical utility of qPCR and ddPCR is very high, and both methods could be recommended for the routine detection of the V617F mutation at diagnosis, though ddPCR will probably supersede qPCR in the future due to cost-effectiveness.

## Introduction

The *JAK2* V617F mutation is present in a high proportion of patients diagnosed with Philadelphia-negative myeloproliferative neoplasms (MPNs). Mutated V617F alleles are detected in approximately 95% of patients diagnosed with polycythemia vera (PV) and in approximately 60% of patients diagnosed with primary myelofibrosis (PMF) and essential thrombocythemia (ET) [1]. Mutations localized within the exon 12 of the *JAK2* gene are rare and described in 2–5% of PV patients [2]. Detection of the *JAK2* V617F mutation is part of the diagnostic criteria of myeloid neoplasms in the recent World Health Organization (WHO) classification [3]. Available data show that the burden of *JAK2* V617F alleles could correlate with the phenotypic presentation of MPN [4], severity of the disease phenotype [5], the risk of thrombotic events [6, 7], progression to post-PV myelofibrosis [8], and survival [9]. Thus, quantification of the *JAK2* V617F mutation at diagnosis provides important prognostic information. In addition, *JAK2* V617F quantification is also very useful during the course of treatment to monitor response to therapy with JAK2 inhibitors, alpha-interferon, or allogeneic stem-cell transplantation (allo-HSCT) [10].

Two highly sensitive molecular techniques—quantitative polymerase chain reaction (qPCR) and droplet digital PCR (ddPCR)—are currently available for a quantitative evaluation of *JAK2* V617F mutation allele burden in patients diagnosed with MPNs. However, the amount of data regarding a direct comparison of these methods used at diagnosis and for minimal residual disease (MRD) monitoring in MPN patients is limited.

## Aims

The aim of this study was to compare the accuracy and clinical utility of qPCR and ddPCR applied at diagnosis and in MRD monitoring of *JAK2* V617F-positive patients with myeloproliferative neoplasms.

## Materials and methods

### Patient samples

Peripheral blood samples were collected from 63 MPN patients at time of diagnosis at the Department of Hematology, University Hospital in Kraków, Poland. The group consisted of 20 patients suffering from PV, 20 patients with ET, and 23 patients with PMF. From six patients diagnosed with PMF undergoing therapy with ruxolitinib (JAK2 inhibitor), an additional blood sample was collected for MRD monitoring. MPNs were diagnosed according to 2008 WHO classification criteria. All patients provided informed written consent, and the study was approved by the local Ethics Committee. Clinical and laboratory characteristics of patients are described in Table 1.

**Table 1:** Clinical characteristics of patients suffering from Ph-negative MPNs at diagnosis. *PV* polycythemia vera, *ET* essential thrombocythemia, *PMF* primary myelofibrosis

Characteristics	PV	ET	PMF
Number of patients	20	20	23
Median age, years (range)	57.5 (20–85)	42.5 (20–82)	62 (35–78)
Gender, M/F	11/9	8/12	13/10
Median hemoglobin, g/dl (range)	16.9 (14–22.7)	13.9 (10.5–16.1)	11.6 (8.2–16)
Median hematocrit, % (range)	52 (40–67)	40.6 (34.1–46.4)	35.9 (28–51)
Median red blood cell, M/μl (range)	5.8 (3.9–7.8)	4.6 (3.5–5.7)	4.2 (2.7–5.9)
Median white blood cell, K/μl (range)	11.1 (4.1–70.2)	7.2 (4.7–19.5)	9.5 (5.2–22.5)
Median platelets, K/μl (range)	694.5 (59–2500)	397 (150–893)	530 (119–1200)
Splenomegaly, number of patients	7 (1 lack of result)	2 (1 lack of result)	12 (2 lack of results)

### Control samples

Two separate groups of control samples were used in the study. For qPCR, blood samples were collected from 30 healthy individuals recruited from the Department of Hematology, University Hospital in Kraków, Poland. For ddPCR, samples from more than 600 normal donors were analyzed, and in 192 samples, positive *JAK2* V617F droplets were detected. The samples were collected at Zealand University Hospital in Roskilde/Naestved, Denmark.

## Methods

### DNA isolation

Blood samples were treated with NH_4_Cl lysis buffer before isolation of nucleic acids. Genomic DNA was extracted from the leukocytes using the NucleoSpinTissue Columns (MACHEREY-NAGEL GmbH & Co. KG, Düren, Germany) according to the manufacturer’s instructions and was quantified spectrophotometrically using a NanoDrop Lite Spectrophotometer (Thermo Fisher Scientific, Wilmington, USA).

### Quantitative PCR (qPCR)

The qPCR was performed as described by Larsen et al. [11]. This assay was found to have the best performance profile and was selected by ELN/MPN&MPNr-EuroNet as the optimal qPCR assay for routine diagnosis and monitoring of MRD in *JAK2* V617F-positive MPN patients [12]. PCR was performed using 25 ng DNA in a total volume of 25 μl (12.5 μl TaqMan Universal PCR Master Mix (with UNG and ROX) [Thermo Fisher Scientific, Waltham, MA USA], 2.5 μl 10× primers/probe mix, 5 μl nuclease-free water, and 5 μl DNA 5 ng/μl). The primer concentration was 300 nmol/l and the probe concentration was 200 nmol/l. The PCR protocol was an initial enzyme activation step of 2 min at 50 °C and 10 min at 95 °C, followed by 50 cycles of 95 °C for 15 s and 60 °C for 60 s. All qPCR reactions were performed in duplicates on the Viia 7 Real-Time PCR System (Applied Biosystem, Foster City, CA). The percentage of *JAK2* V617F mutated alleles were calculated as [copy-number_*JAK2*V617F_/(copy-number_*JAK2*V617F_ + copy-number_*JAK2*wild-type_)] × 100. This calculation was made with the use of qPCR standard curve in which the absolute copy number of mutated allele and wild-type allele of *JAK2* in each standard sample was evaluated previously by ddPCR. The standard curve was prepared following recommendations of MPN&MPNr-EuroNet consortium as a result of quality control assurance of qPCR methods for *JAK2*V617F evaluation.

### Droplet digital PCR (ddPCR)

The Bio-Rad’s QX200 system (Bio-Rad Laboratory, Hercules, California, USA) was used to perform ddPCR. In each ddPCR reaction, two probes were used: a FAM-labeled probe for the *JAK2* V617F mutation and a HEX-labeled probe for the *JAK2* V617F wild-type allele. Usage of two probes in one reaction allowed the simultaneous detection and calculation of both the mutant and the wild-type alleles. The reaction volume was 20 μl (10 μl 2× ddPCR Supermix for Probes [no dUTP], 2 μl 10× primers/probes mix, 3 μl nuclease-free water with 5 μl of genomic DNA). The primer concentration was 300 nmol/l, and the probe concentration was 200 nmol/l. PCR was performed in a Veriti PCR instrument (Thermo Fisher) using the following conditions: 95 °C for 10 min, followed by 40 cycles of 94 °C for 30 s, and 57 °C for 60 s with a final stage at 98 °C for 10 min. After thermal cycling, the 96-well plate was read in the QX200 Droplet Reader, and based on positive droplets and according to the Poisson distribution, the absolute copy number of the *JAK2* V617F and wild-type *JAK2* alleles was calculated using the QuantaSoft analysis software (Bio-Rad Laboratory, Hercules, California, USA). The percentage of *JAK2* V617F mutated alleles were calculated as [copy-number_*JAK2*V617F_/(copy-number_*JAK2*V617F_ + copy-number_*JAK2*wild-type_)] × 100. The samples were analyzed in triplicates.

### Evaluation of the limit of blank (LoB) and the limit of detection (LoD)

Limit of blank (LoB) and limit of detection (LoD) were calculated to avoid false positive and false negative results, and reliably to measure and critically describe the minimum amount of *JAK2* V617F that could be detected in the context of highly sensitive allele-specific assays.

Both parameters for qPCR and ddPCR platforms were independently tested and based on different groups of samples.

### LoB and LoD for qPCR

For the qPCR method, the LoB was established by measuring false positive events from a series of 30 wild-type control samples. The calculation was based on the average value of the measurements using the formula: LoB = mean blank + 1645 × SD_blank_, where SD is the standard deviation. By the estimation of LoB, the lowest *JAK2* V617F allele burden with 95% confidence was set with clearly cut-off signals from the background.

LoD was established by preparation of twofold dilution series of genomic DNA derived from UKE-1 cell line (homozygous *JAK2* V617F mutation) re-suspended in wild-type DNA, covering the range 0.0098–2.5000% of *JAK2* V617F per reaction volume. Each standard point was analyzed in 50 replicates. To identify the outliers, Grubb’s test was performed, and finally, 444 data points were used for the analysis. The received data were analyzed using GeneEx 6.1 (trial version, MultiD analyses AB) under current MIQE (Minimum Information for Publication of Quantitative Real-Time PCR Experiments) [13] and CLSI (Clinical Laboratory Standards Institute) guidelines [14] according to the protocol described by Forootan et al. [15]. LoD gives the possibility to detect the lowest percentage of *JAK2* V617F allele burden in a sample with 95% probability, ensuring ≤ 5% false positives, in the used molecular method. Furthermore, the limit of detection accurately expresses the analytical sensitivity of an assay [13].

### LoB and LoD for ddPCR

The calculation of LoB was based on the average percentage of *JAK2* V617F mutated alleles measured in the 192 normal donors samples, where *JAK2* V617F-positive droplets were detected. The LoD was calculated with the 99% confidence from the formula: LoD = LoB + 3 × SD.

### Statistical analysis

The GraphPad version 4.0 software (La Jolla, CA, USA) was used to calculate the statistical parameters. A comparison between qPCR and ddPCR, on the basis of clinical samples, was performed using the Spearman rank correlation coefficient analysis. Bland-Altman analysis was used to evaluate the agreement among the two PCR methods in clinical samples. Subsequently, a comparison between allele burden medians measured by both methods separately for different MPNs was performed by the Mann-Whitney test. Additionally, six patients were compared before and after 2–22 months of therapy with ruxolitinib, and the quantitative variable was analyzed with the Student paired *t* test. A *p* value < 0.05 was considered statistically significant.

## Results

**Fig. 1:**
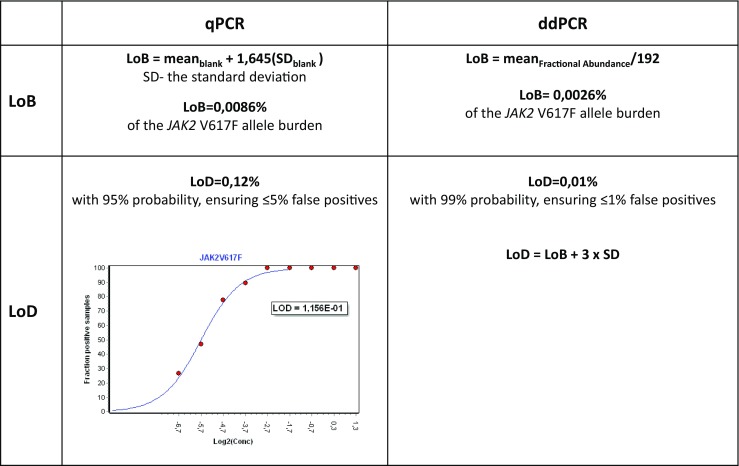
Estimation of limit of detection (LoD) for qPCR and ddPCR methods. For qPCR, fractions of positive reads were fitted to sigmoidal curve and the LoD was estimated at 95% confidence level. For ddPCR, LoD calculation was based on limit of blank (LoB) value and standard deviation (SD) of measurements

The calculation of LoB for qPCR defines background level based on the 30 wild-type control samples and was equaled to 0.0086% of the V617F allele burden. For the ddPCR, the calculation of LoB was determined to 0.0026%. Considering the assay performance, the established LoD defined the lowest concentration of *JAK2* V617F allele that could be detected and was set at 0.12% for qPCR (95% confidence level), and 0.01% for ddPCR (99% confidence level) (Fig. 1). These data showed one logarithm higher sensitivity of ddPCR compared to qPCR.

**Fig. 2:**
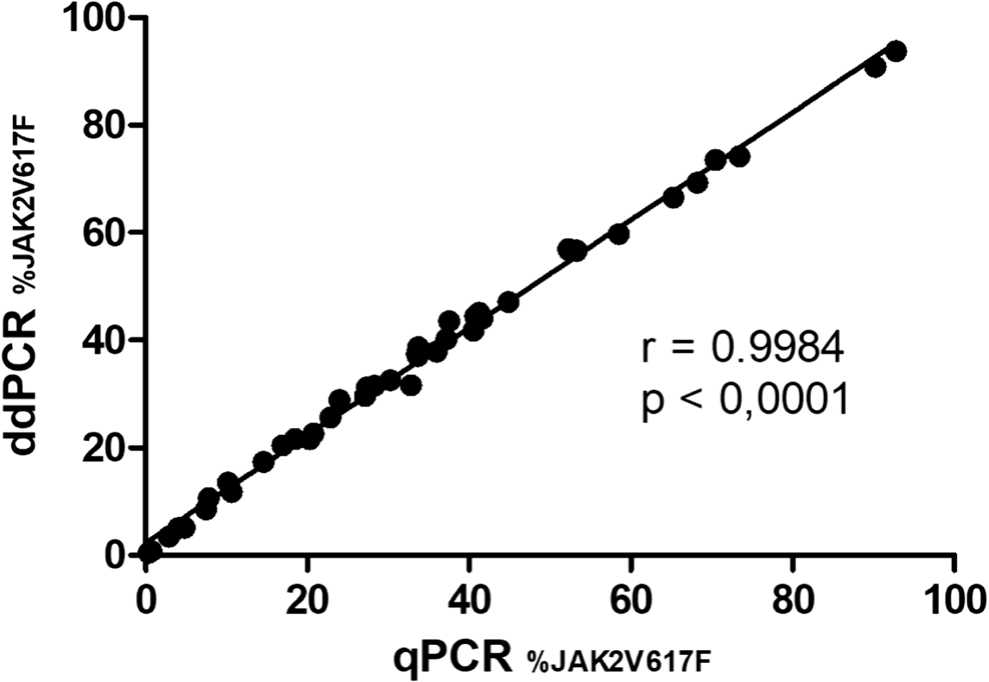
Correlation between qPCR and ddPCR (*r* = spearman correlation coefficient)

**Fig. 3:**
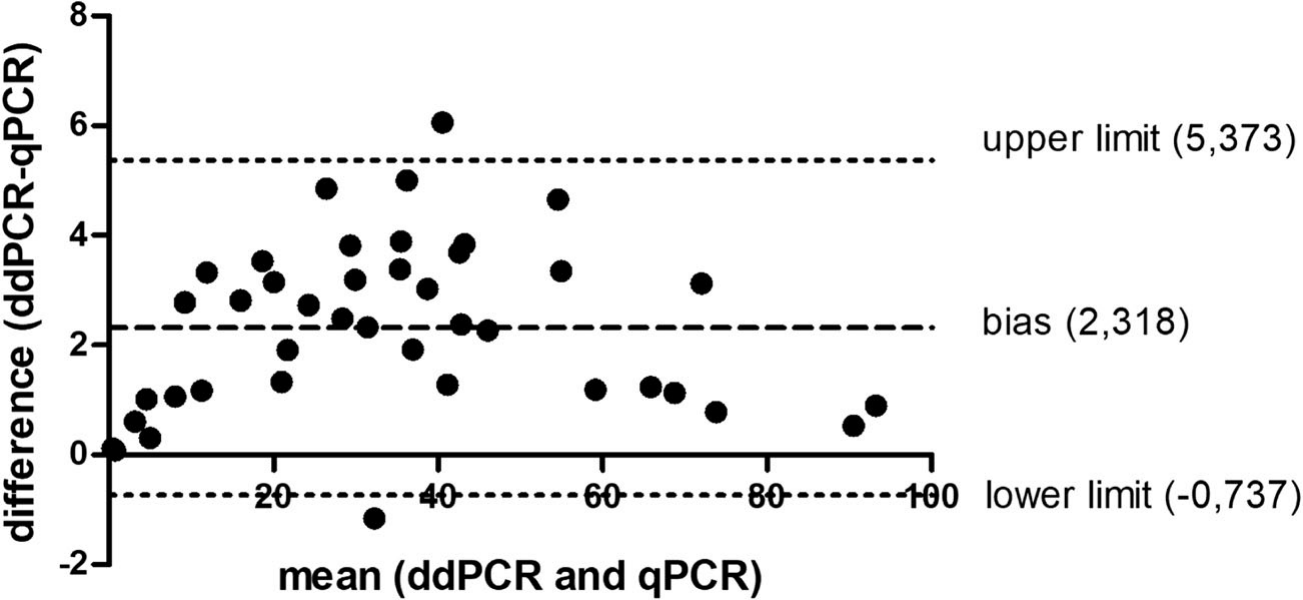
The differences between measurements by qPCR and ddPCR (*y* axis) vs. the mean of the sample quantification performed by qPCR and ddPCR (*x* axis) (the Bland-Altman plot). Dotted lines indicate bias (mean difference) and 95% limits of agreement (upper limit + 2 SD; lower limit − 2 SD) between the two given methods. A 95.12% of presented measurements fitted to the limit of agreement

**Fig. 4:**
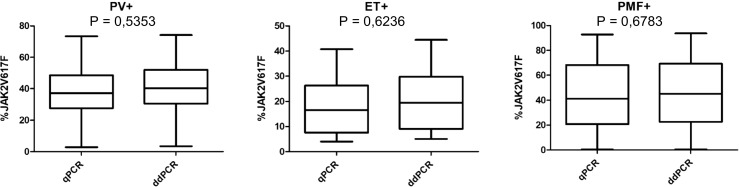
Comparison of median *JAK2* V617F allele burden measured by qPCR and ddPCR. Mann-Whitney *U* test shows no significant differences between the two methods. PV+: patients diagnosed with polycythemia vera, ET+: patients diagnosed with essential thrombocythemia, PMF+: patients diagnosed with primary myelofibrosis

The Spearman’s rho test showed a high conformance between the two methods with a correlation coefficient of *r* = 0.998, *p* < 0.0001 (Fig. 2). The test confirmed 100% positive results overlap between the methods. Additionally, in a Bland-Altman test, the bias between the measurements obtained by qPCR vs ddPCR was evaluated. The bias was at the level of 2.3 and more than 95% (95.15%) of differences of measurements lay within the limit of agreement (Fig. 3). Finally, the medians of the *JAK2* V617F quantification were comparable between the two methods (Fig. 4).

**Fig. 5:**
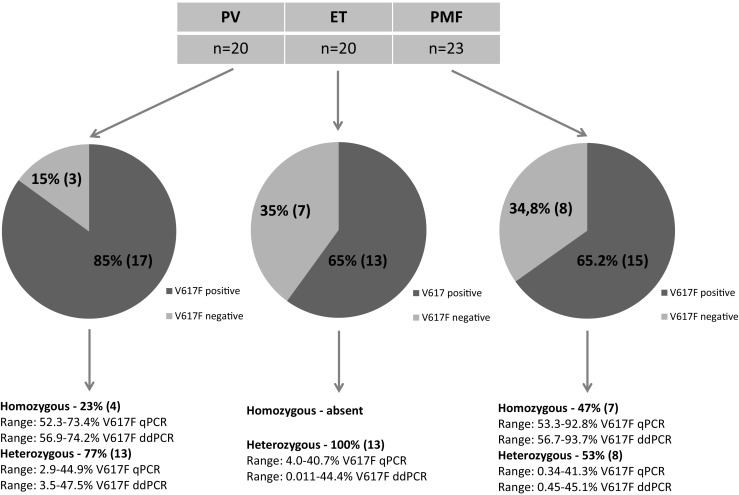
Distribution of *JAK2* V617F-positive MPN patients and their allele burden within subgroups on the basis of ddPCR results. Among the patients diagnosed with ET, the *JAK2* V617F mutation was detected in 12 patients (60%) with qPCR and in 13 patients (65%) with ddPCR. One patient had a low mutation burden of 0.011% measured by ddPCR with a corresponding mutation burden of 0.02% measured in qPCR (this value was calculated below qPCR LoD)

Among the 63 patients diagnosed with MPNs, the *JAK2* V617F mutation was detected using qPCR in 44 patients (69.8%). The median allele burden was 33.2% (range 0.34–92.8%). In ddPCR, 45 of 63 patients (71.4%) were positive for *JAK2* V617F mutation with a median allele burden of 36.5% (range 0.011–93.7%). The mutation was identified in 85% of patients with PV. The median allele burden was 37.2% (range 2.9–73.4) according to qPCR analysis. In ddPCR, the median allele burden was 40.2% (range 3.5–74.2%). Additionally, in both methods, four PV patients were characterized as homozygous, 13 as heterozygous, and in 3 (15%) patients, the *JAK2* V617F mutation was not detected (including mutations in exon 12). The *JAK2* V617F-negative patients with erythrocytosis was diagnosed with PV according to 2008 WHO classification criteria and after thorough analysis of trephine biopsy. Among ET patients, 12 (60%) and 13 (65%) were tested *JAK2* V617F mutation positive in qPCR and ddPCR, respectively. The median allele burden was 16.5% (range 4.0–40.7%) by qPCR and 17.4% by ddPCR (range 0.011–44.4%), respectively. One ET patient had a low mutation burden of 0.011% measured by ddPCR. With a corresponding mutation burden by qPCR calculated to be below LoD: 0.02%, the *JAK2* V617F positivity remains inconclusive in this patient. In the ET group, all positive patients were heterozygous. The measurements in PMF patients showed the presence of the mutation in 65.2% cases both by qPCR and ddPCR. The median allele burden by qPCR was 37.5% (range 0.34–92.8%) and 42% (range 0.45–93.7%) by ddPCR. Both methods confirmed the presence of homozygosity in seven patients and heterozygosity in eight patients (results demonstrated in Fig. 5).

No statistical differences between qPCR and ddPCR in the assessments of *JAK2* V617F allele burden within all three subgroups of MPNs were found (Fig. 4). Of note, despite no statistical difference were revealed, ddPCR values were slightly higher than qPCR in all but two cases (0.002% and 0.011% for ddPCR and 0.010% and 0.02% for qPCR, respectively).

The allele burdens in the six PMF patients at diagnosis compared with the allelic burden during ruxolitinib therapy showed statistically significant differences when measured with ddPCR, whereas this was seen in five patients measured with qPCR. In four out of six patients, we observed a statistically significant reduction of the mutated allele burden during ruxolitinib therapy (Table 2). According to the revised IWG-MRT and ELN recommendations, patients 2–6 fulfilled criteria for partial molecular remission assessment (≥ 12-week time interval between allele burden measurements and a mutant allele burden at baseline ≥ 20%). However, none of the analyzed patients obtained a partial molecular remission (≥ 50% decrease in allele burden). In two patients (no. 3 and no. 5), the allele burden increased during therapy (Table 2) which corresponded to progressive splenomegaly and increased percentage of myeloblasts in peripheral blood suggesting the progression of the disease.

**Table 2:** V617F mutant allele load analyses in patients with PMF treated with JAK2 inhibitor (ruxolitinib)

		% JAK2V617F		Nominal allele burden change	*p* value (*p < 0.05)	Relative allele burden change	% JAK2V617F		Nominal allele burden change	*p* value (*p < 0.05)	Relative allele burden change
		Before JAK2 inhibitor therapy	During JAK2 inhibitor therapy				Before JAK2 inhibitor therapy	During JAK2 inhibitor therapy			
1	2	0.61	0.03	− 0.58	0.0296*	95.1%	0.75	0.069	− 0.68	0.0025*	90.8%
2	22	55.45	42.92	− 12.53	0.0071*	22.6%	56.66	42.62	− 14.04	0.001*	24.8%
3	19	66.60	75.67	+ 9.07	0.0184*	13.6%	66.5	77.39	+ 10.89	0.0196*	16.4%
4	3	29.96	20.53	− 9.43	0.0106*	31.5%	36.46	21.47	− 14.99	0.0026*	41.1%
5	3	73.41	74.68	+ 1.27	0.665	1.7%	75.42	78.15	+ 2.73	0.0151*	3.6%
6	3	65.12	60.14	− 4.98	0.0016*	7.6%	68.98	62.32	− 6.66	0.0007*	9.6%

## Discussion

The prognostic significance of the *JAK2* V617F mutation, including the allelic burden, has been investigated in many studies [6, 7, 16, 17]. In our study, we compared two highly sensitive molecular methods: the qPCR and ddPCR in relation to detection and quantification of the *JAK2* V617F mutation in patients suffering from MPNs. We detected the *JAK2* V617F mutation to be present in about two-thirds of our ET and PMF patients, which is in line with other reports [7, 9, 18–21]. The percentage of *JAK2* V617F mutation detected in our PV patient group was lower (85%) than is usually reported [5, 21–23], probably due to a limited number of samples analyzed. Homozygosity in PV and ET has been associated with increased risk of fibrotic transformation [17, 24–27], lower platelet count, higher incidence of splenomegaly, and larger spleen size [16, 17]. Homozygous ET patients also have a higher incidence of thrombotic events [24]. In accordance with other studies [4, 26], our quantitative measurements of PV and PMF patients revealed a higher median *JAK2* V617F allele burden compared to ET patients. Homozygosity in ET patients only occurs rarely in up to 2–4% of cases [6, 16], and accordingly, none of our ET samples showed a mutational load higher than 50% (Fig. 5). These results show a concordance between the level of allele burden and severity of MPN disease in that PV and PMF have a poorer overall survival than ET [28]. In contrast, a low allele burden within PMF patients has been associated with more severe disease resulting in a reduced overall survival, which is probably due to a cytopenic phenotype rather than a myeloproliferative one [7]. Additionally, it has been suggested that a level of mutation ≥ 50% in PMF predicts a better response to JAK2 inhibitor therapy [29]. In addition to be this “genetic risk factor” for prognosis, *JAK2* V617F allele burden may also be used as a MRD tool. According to the Italian Society of Hematology, high-sensitivity quantitative assays are recommended for monitoring MRD in all PMF patients after allogenic stem cell transplantation (allo-SCT) [10] in order to identify patients at higher risk of relapse [30] with the need of preemptive donor lymphocyte infusion immunotherapy [31].

In our study, both qPCR and ddPCR showed consistent and statistically important differences in the level of mutation before and during therapy with ruxolitinib in six patients. However, due to relatively short-term follow-up, no prognostic conclusion could yet be drawn. International Working Group-Myeloproliferative Neoplasms Research and Treatment (IWG-MRT) and the European LeukemiaNet recently proposed molecular remission as one of the response criteria of myelofibrosis therapy, which emphasizes the importance of accurate and sensitive methods in the era of novel and more effective therapies in PMF. It has been proposed that when screening for *JAK2* V617F mutation, the qPCR should be the method of choice due to a greater reproducibility and sensitivity compared to qualitative methods, and for molecular monitoring of MRD after allo-SCT, ddPCR would be a preferred option [10]. In the latter case, the ability to detect mutated clones even with low allele burden (mutation loads < 1%) allows to predict relapse [12]. According to Watherhouse et al., *JAK2* V617F mutation was detected with higher frequency by ddPCR than qPCR. Digital PCR allows good performance by achieving absolute quantification, thereby minimalizing the difficulties of result interpretation when test results are below the limit of quantification and over the limit of detection [32].

In patients suffering from MPNs, the positivity threshold for quantitative detection of *JAK2* V617F mutation at diagnosis should be at least 1–3% of allele burden [33–35]. In our study, both qPCR and ddPCR demonstrated high sensitivity, enabling to detect the mutant allele burden at diagnosis, also below 1%. The information regarding the size of the mutated clone at diagnosis could be very important, since even low allele burden could be pathologically relevant and clinically significant. The implementation of ddPCR in the diagnostics of MPN could result in detection of MPN at early stage. The preliminary results, obtained on 20,000 healthy Danish people, consider the low allele burden less than 1% as correlating with mild leukocytosis. This in future perspective may result in reduction of thrombotic and hemorrhagic events in patients, due to earlier diagnosis of pre-MPN and introduction of proper treatment [36]. The measurement of the low *JAK2* V617F allele burden could offer the new prognostic parameter, which presumably could be applied for therapeutic interventions. Another aspect of using ddPCR method with higher LoD in detection of *JAK2* V617F mutation is the association with clonal hematopoiesis of indeterminate potential (CHIP). The presence of genetic abnormalities in genes related to the development of myeloid neoplasms in aging population (e.g., *JAK2*, *DNMT3*, *TET2*, and *ASXL1*) [37] is associated with increased risk of hematologic malignancies, all-cause mortality, and cardiovascular disease [37, 38]. In these cases, the evaluation of low *JAK2* V617F allele burdens in the healthy individuals in contribution with other CHIP mutations could serve as an important marker for early detection of clinical progression to disease in at-risk individuals [39, 40]. Furthermore, ddPCR could detect low allele burdens as a sign of the presence of a small mutated clone within an overall polyclonal hematopoiesis [41], which may arise independently [41, 42] and finally lead to myeloproliferative disease. Therefore, monitoring of low *JAK2* V617F allele burden by ddPCR over time with clinical observation could become an important strategy. Furthermore, in these cases, further screening for the coexistence of mutation occurring either in *JAK2* exon 12, in *MPL* or in *CALR* gene is recommended [41, 43, 44]. In our study, in all three patients with low *JAK2* V617F allele burden (< 1%) detected by qPCR and ddPCR, additional analyses showed a complex mutational pattern with coexistence of mutations in the *ASXL1*, *CALR*, and *MPL* gene, respectively (data not shown). However, the detection of small clones around and below 1% should be considered in the context of other clinical parameters, since the V617F mutation of *JAK2* gene has been detected at low levels in other disorders than MPNs as well as in healthy individuals, and its clinical significance is still not established [33–35]. Therefore, in our study, a non-specific background below the LoB for qPCR and ddPCR methods was estimated based on healthy controls. Furthermore, the threshold for false positive events was estimated independently for qPCR and ddPCR which allowed to classify wild-type samples as negative by both methods.

The high quality of the qPCR assay used within this study for *JAK2* V617F quantification was confirmed during quality control assurance conducted by MPN&MPNr-EuroNet consortium. A corresponding international quality control and an assay standardization have not yet been established for ddPCR. Therefore, the application of ddPCR in diagnostic and treatment evaluation has been based on clinical and methodological experiences developed in-house. Clinical-diagnostic assays require high-throughput protocol with accurately fixed analytical parameters such as LoD. Our experiments revealed high analytical sensitivity and specificity for both tests, suggesting that they could serve as reliable diagnostic tools to detect the *JAK2* V617F mutation in the diagnosis of MPN. Our comparison of qPCR and ddPCR suggests that the two methods could be used interchangeably for diagnostic purposes and MRD monitoring, provided that the clinically acceptable discrepancy between the results falls within the designated agreement interval. Although the bias between the methods is relatively small, its clinical relevance should be, however, taken into account in patients with low allele burdens, such as during evaluation of treatment response and/or relapse after allo-SCT. In some rare cases, however, none of the assays can guarantee 100% accurate quantification of mutation alleles. The co-existence of additional mutation within the primer or probe-annealing site can potentially reduce or prevent the amplification of the investigated sequence [34].

It should be emphasized that ddPCR is characterized by higher precision and sensitivity in assessment of a low concentration of the *JAK2* V617F mutation, which is important in terms of measuring the actual biological and clinical mutation status [31]. Therefore, ddPCR seems to be particularly suitable for precise monitoring of MRD. Last but not least, the ddPCR method is more cost-effective than qPCR since it is a multiplex reaction in contrast to two independent qPCR reactions with the need for running additional standard curves. Our findings suggest that ddPCR could be a complementary approach for MRD monitoring in MPN patients and that ddPCR probably will supersede qPCR in the future.
